# Genetic diversity into a novel free-living species of *Bradyrhizobium* from contaminated freshwater sediment

**DOI:** 10.3389/fmicb.2023.1295854

**Published:** 2023-11-18

**Authors:** Naxue Zhang, Chun-Zhi Jin, Ye Zhuo, Taihua Li, Feng-Jie Jin, Hyung-Gwan Lee, Long Jin

**Affiliations:** ^1^College of Ecology and Environment, Nanjing Forestry University, Nanjing, China; ^2^Cell Factory Research Centre, Korea Research Institute of Bioscience and Biotechnology (KRIBB), Daejeon, Republic of Korea; ^3^Department of Environmental Biotechnology, KRIBB School of Biotechnology, Korea University of Science and Technology (UST), Daejeon, Republic of Korea

**Keywords:** *Bradyrhizobium*, *Bradyrhizobium roseus*, free-living, non-symbiotic, phosphonate, freshwater sediment

## Abstract

A free-living *Bradyrhizobium* strain isolated from a contaminated sediment sample collected at a water depth of 4 m from the Hongze Lake in China was characterized. Phylogenetic investigation of the 16S rRNA gene, concatenated housekeeping gene sequences, and phylogenomic analysis placed this strain in a lineage distinct from all previously described *Bradyrhizobium* species. The sequence similarities of the concatenated housekeeping genes support its distinctiveness with the type strains of the named species. The complete genome of strain S12-14-2 consists of a single chromosome of size 7.3M. The strain lacks both a symbiosis island and important nodulation genes. Based on the data presented here, the strain represents a new species, for which the name *Bradyrhizobium roseus* sp. nov. is proposed for the type strain S12-14-2*^T^*. Several functional differences between the isolate and other published genomes indicate that the genus *Bradyrhizobium* is extremely heterogeneous and has functions within the community, such as non-symbiotic nitrogen fixation. Functional denitrification and nitrogen fixation genes were identified on the genomes of strain S12-14-2*^T^*. Genes encoding proteins for sulfur oxidation, sulfonate transport, phosphonate degradation, and phosphonate production were also identified. Lastly, the *B. roseus* genome contained genes encoding ribulose 1,5-bisphosphate carboxylase/oxygenase, a trait that presumably enables autotrophic flexibility under varying environmental conditions. This study provides insights into the dynamics of a genome that could enhance our understanding of the metabolism and evolutionary characteristics of the genus *Bradyrhizobium* and a new genetic framework for future research.

## Introduction

The genus *Bradyrhizobium* is a relatively well-studied rhizobial bacteria and well known for symbiosis and endophytic interaction with leguminous plants, where they exchange nutrients for survival. The genus includes a diverse collection of globally distributed bacteria that can nodulate a wide range of legumes (Salminen and Streeter, 1987; Van Rhijn and Vanderleyden, 1995; Okubo et al., 2012; Parker, 2015; Remigi et al., 2016; Sprent et al., 2017; Claassens et al., 2023; Lafay et al., 2023). It was widely recognized that the *Bradyrhizobium* species were capable of nitrogen fixation and the formation of symbiotic nodules in the roots of leguminous plants. The symbiotic species are crucial for soil health, plant growth, and food security, whereas nitrogen-fixing species are critical for the global nitrogen cycle (Jordan, 1982; Ladha and So, 1994; Stacey et al., 1995; Remigi et al., 2016; Gogoi et al., 2018). Members of *Bradyrhizobium* are characterized extensively for their symbiotic lifestyle with leguminous plants to form symbiotic nodules. *Bradyrhizobium* has biologically significant functions in soils, including photosynthesis, nitrogen fixation, denitrification, and degradation of aromatic compounds and others. Especially in the global nitrogen cycle, *Bradyrhizobium* removes nitrogen through heterotrophic denitrification and also has multiple roles in agriculture (Kaneko et al., 2002; Okubo et al., 2012). Nodulating *Bradyrhizobium* species typically contain *nod* and *nif* genes associated with nodulation and nitrogen fixation, respectively, the so-called symbiotic properties. Due to these characteristics, *Bradyrhizobium* has been regarded as a model of legume-rhizobia symbiosis and an ecologically significant microbe. However, nodulation-deficient isolates have been discovered and characterized, even though they possess genes for nitrogen fixation, and some *Bradyrhizobium* isolates recovered from forest soils have been found to lack both nodulation and nitrogen-fixing functions (VanInsberghe et al., 2015; Jones et al., 2016). A culture-independent investigation has shown that the population of *Bradyrhizobium* in soil habitats is different from the rhizosphere of leguminous plants (VanInsberghe et al., 2015), suggesting that non-symbiotic *Bradyrhizobia* inhabit physically or functionally distinct niches.

Understanding the mechanisms of bradyrhizobial adaptation to independent existence in diverse environments is becoming increasingly important and urgent and may reveal the genetic potential of this globally significant genus. In the present study, we characterized a novel species of *Bradyrhizobium* isolated from freshwater sediment of the Hongze Lake in China. To reveal the genetic diversity and physiological features, here, we report the isolation, and the sequencing, assembly, taxonomic classification, and annotation of the genome of a new species of the genus *Bradyrhizobium*. These investigations will be extremely beneficial for elucidating its putative metabolism, contributing to a greater understanding of free-living *Bradyrhizobium* isolates, enhancing knowledge of the genes and enzymes involved in metabolic pathways, and possibly identifying genomic markers that can be utilized in future ecological studies.

## Materials and methods

### Sampling, isolation, and growth condition

Strain S12-14-2 was recovered from a contaminated sediment sample (33°14′26″N, 118°35′40″E) collected at a water depth of 4 m from Hongze Lake, the fourth largest freshwater lake in China. The Hongze Lake is located in the Jiangsu Province and surrounded by Suqian and Huai’an cities; it has a surface area of 1,960 km^2^, a mean water depth of 1.77 m, a maximum depth of 4.37 m, and a volume of 28 × 108 m^3^ (Gao et al., 2009; Li et al., 2011). The ecological risk of the Hongze Lake sediments has been assessed. Heavy metals including copper (Cu), zinc (Zn), lead (Pb), cadmium (Cd), chromium (Cr), mercury (Hg), arsenic (As), iron (Fe), aluminum (Al), and manganese (Mn) have been identified in the lake sediments of the Hongze Lake, and antibiotics like atrazine and ofloxacin were also detected (Yao et al., 2016; Wu et al., 2023). One gram of a sediment sample was used to screen for bacterial strains with the serial dilution method. The strain was grown on modified R2A medium at 30°C under dark conditions (Jin et al., 2019).

### Morphological and physiological characteristics

The mobility and morphology of the colonies were examined using a phase-contrast microscope (Nikon Optiphot, 1,000× magnification) and transmission electron microscopy (Philips CM-20). The growth temperature range, pH range, and salt tolerance were determined following the methods described by Jin et al. (2021). Duplicated antibiotic-susceptibility of strain S12-14-2 was checked on R2A agar medium using the filter-paper disk method (Li et al., 2020), where the disks contained the following: nalidixic acid (30 μg ml^–1^), tetracycline (30 μg ml^–1^), amikacin (30 μg ml^–1^), ampicillin/sulbactam (20 μg ml^–1^, 1:1), kanamycin (30 μg ml^–1^), vancomycin (30 μg ml^–1^), chloramphenicol (30 μg ml^–1^), teicoplanin (30 μg ml^–1^), spectinomycin (25 μg ml^–1^), gentamicin (30 μg ml^–1^), streptomycin (25 μg ml^–1^), rifampicin (30 μg ml^–1^), lincomycin (15 μg ml^–1^), and erythromycin (30 μg ml^–1^). Positive results were observed for halo diameters greater than 10 mm after 5 days of incubation at 30°C. For the comparative cellular fatty acids analysis, strain S12-14-2 together with the reference strains *Bradyrhizobium erythrophlei* LMG 28425*^T^*, *Bradyrhizobium jicamae* LMG 24556*^T^*, *Bradyrhizobium lablabi* LMG 25572*^T^*, *Bradyrhizobium mercantei* LMG 30031*^T^*, *Bradyrhizobium elkanii* KACC 10647*^T^*, and *Bradyrhizobium japonicum* KACC 10645*^T^* were cultured on R2A agar for 2 days at 30°C. Standardized cell harvesting and extraction were performed according to Jin et al. (2022a), and the identification was performed using the TSBA 6 (Trypticase Soy Broth Agar) database with the Sherlock software 6.1. Carbon source utilization, enzyme activities and other physiological and biochemical activities were observed using the API 20NE, API ID 32GN, and API ZYM kits (bioMérieux, l’Etoile, France) according to the manufacturer’s instructions.

### DNA extraction, PCR amplification, and genomic sequencing

Genomic DNA was extracted using the FastDNA™ SPIN kit following the manufacturer’s instructions. The concentration and purity of the DNA were then checked on a ND-2000 spectrophotometer (Thermo Fisher Scientific). The 16S rRNA genes of strain S12-14-2 were amplified by PCR method using the universal primer sets 27F/1492R (Weisburg et al., 1991) and then identified using the EzBioCloud web service (Yoon et al., 2017). The use of multilocus sequence analysis (MLSA) has been proven useful for the classification and identification of several groups of rhizobia. In the genus *Bradyrhizobium*, the 16S rDNA sequences provide minimal taxonomic information due to the high degree of conservation among species and the relatively high sequence similarity. Consequently, MLSA studies of *glnII*, *recA*, *dnaK*, *rpoB*, and ITS genes were performed to assess the taxonomic position of the free-living strain among identified species (Martens et al., 2008; Menna et al., 2009; Rivas et al., 2009; Pérez-Yépez et al., 2014; Gnat et al., 2016). The partial sequences of the housekeeping genes, *glnII*, *recA*, *dnaK*, *rpoB*, and ITS genes, were amplified by PCR method. The primer sets and PCR amplification conditions were described in a previous study (Menna et al., 2009; Delamuta et al., 2013). Whole genome sequencing was done at Novogene Biotechnology, Beijing, China, using the Pacific Biosciences (PacBio) RS II single molecule real-time (SMRT) platform together with the Illumina HiSeq technology. The quality of the genome assemblies was assessed using SMRT Link version 5.0.1.

### Phylogenetic analyses and genome annotation

For phylogenetic analysis of strain S12-14-2, the 16S rRNA sequences of *Bradyrhizobium* type strains were downloaded from the NCBI database. The sequences were edited and aligned with BIOEDIT and CLUSTAL X software, respectively (Thompson et al., 1997; Hall, 1999), and the phylogenetic tree was reconstructed using the MEGA7 software (Kumar et al., 2016). For genomic annotations, the draft genome sequence was applied to the RAST pipeline, and comparisons of the genome were performed in the SEED Viewer (Aziz et al., 2008, 2012). The protein coding sequences (CDSs) were submitted to the COG (Clusters of Orthologous Groups) database^1^ for functional classification and summary statistics (Tatusov et al., 1997, 2003). For the pangenome analysis, we followed Delmont and Eren’s description of the Anvi’o workflow (Delmont and Eren, 2018), a community-driven, open-source analysis and visualization platform for microbial-omics, which is available at http://merenlab.org/software/anvio/. The structures of protein monomeric units were predicted using the SWISS-MODEL (Bienert et al., 2017; Waterhouse et al., 2018). Biosynthetic gene clusters (BGC) were identified and analyzed using the secondary metabolite databases antiSMASH database.^2^ The average nucleotide identity (ANI) was determined using the OrthoANI tool in the EZBioCloud server, and the digital DNA–DNA hybridization (dDDH) value was calculated with the Genome-to-Genome Distance Calculator (GGDC 2.1) (Meier-Kolthoff et al., 2013; Yoon et al., 2017). The average amino acid identity (AAI) was calculated with the AAI web-based calculator developed by Konstantinidis Lab.^3^

## Results and discussion

### Physiological features

The strain displayed growth on R2A and NA agar media after 5 days of incubation at 28°C but not on TSA, LB, or MA media. Colonies were pink in color and less than 1 mm in diameter after 5 days of growth at 28°C on R2A agar medium. Microscopically, the strain represented a Gram-negative and rod-shaped bacterium that ranged in size from 2.0-2.3 × 0.7-0.8 μm (Supplementary Figure 1). Strain S12-14-2*^T^* showed resistance to the following antibiotics tested: nalidixic acid (30 μg ml^–1^), vancomycin (30 μg ml^–1^), chloramphenicol (30 μg ml^–1^), teicoplanin (30 μg ml^–1^), streptomycin (25 μg ml^–1^), rifampicin (30 μg ml^–1^), and lincomycin (15 μg ml^–1^); and susceptible to tetracycline (30 μg ml^–1^), amikacin (30 μg ml^–1^), ampicillin/sulbactam (20 μg ml^–1^, 1:1), kanamycin (30 μg ml^–1^), spectinomycin (25 μg ml^–1^), gentamicin (30 μg ml^–1^), and erythromycin (30 μg ml^–1^). The major fatty acids (>10%) of the strain were sorted into two groups C_16:0_ and C_18:1_ ω7*c* and/or C_18:1_ ω6*c*, which was consistent with species from the genus *Bradyrhizobium* (Supplementary Table 1). The carbon source assimilation, enzyme activity, and some other physiological features are summarized in Supplementary Table 2.

### Genomic analysis: the taxonomic status

**FIGURE 1:**
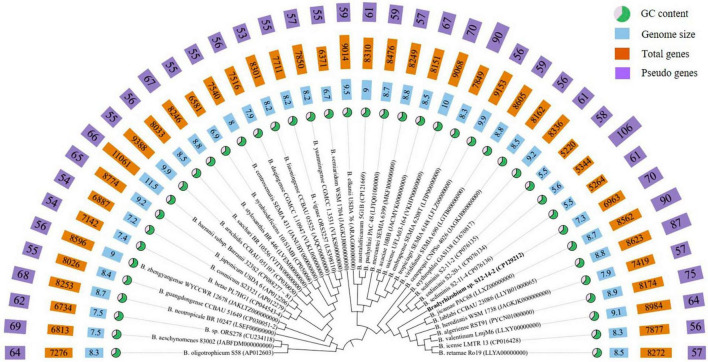
Phylogenomic tree based on genome sequences of strain S12-14-2^T^ and its closely related type strains of *Bradyrhizobium* in the TYGS (https://tygs.dsmz.de/) and iTOL.

The phylogenetic analysis based on 16S rRNA gene sequences revealed that strain S12-14-2 should taxonomically be classified to the genus *Bradyrhizobium*. The strain shared 99.36–99.69% pairwise similarity with *B. lablabi* CCBAU 23086*^T^*, *B. jicamae* PAC68*^T^*, *B. erythrophlei* CCBAU 53325*^T^*, and *B. algeriense* RST89*^T^* and less than 99.3% with the other species within the genus *Bradyrhizobium* (Table 1). The 16S rRNA gene sequence neighbor-joining tree revealed that strain S12-14-2 unambiguously clustered with members of *Bradyrhizobium*. Due to its high level of conservation within the genus *Bradyrhizobium*, the 16S rRNA gene is an inadequate molecular marker for distinguishing species (Germano et al., 2006; Rivas et al., 2009). Therefore, a genome-based phylogenetic analysis was used. The topology of the phylogenetic and phylogenomic trees is consistent with the sequence similarities of the 16S rRNA gene between the novel strain and the type strains of the *Bradyrhizobium* species (Figure 1 and Supplementary Figure 2).

**TABLE 1:** 16S rRNA gene, ANI, AAI similarities (%), and digital DNA–DNA relatedness (%) between novel strain S12-14-2^T^ and related type strains of *Bradyrhizobium*.

No.	Type strains	16S rDNA	ANI	AAI	dDDH
1	*B. sediminis* S2-20-1^T^ (CP076134)	99.3	81.9	80.0	24.9
2	*B. algeriense* RST89^T^ (PYCM00000000)	99.7	86.6	86.5	32.5
3	*B. daqingense* CGMCC 1.10947^T^ (VLKL00000000)	97.7	79.2	75.0	22.8
4	*B. elkanii* USDA 76^T^ (ARAG00000000)	99.0	81.2	76.6	24.7
5	*B. embrapense* SEMIA 6208^T^ (LFIP00000000)	99.3	81.0	76.8	24.1
6	*B. erythrophlei* GAS138 (LT670817)	99.6	79.9	74.6	23.7
7	*B. icense* LMTR 13^T^ (CP016428)	99.3	85.1	85.1	29.5
8	*B. japonicum* USDA 6^T^ (AP012206)	98.2	79.7	74.6	23.0
9	*B. jicamae* PAC68^T^ (LLXZ00000000)	99.4	85.5	85.4	30.0
10	*B. lablabi* CCBAU 23086^T^ (LLYB01000065)	99.4	85.5	71.6	29.8
11	*B. mercantei* SEMIA 6399^T^ (MKFI00000000)	99.3	81.1	76.5	24.5
12	*B. neotropicale* BR 10247^T^ (LSEF00000000)	98.9	79.3	74.4	22.9
13	*B. pachyrhizi* PAC 48^T^ (LFIQ01000000)	99.0	81.1	76.5	24.5
14	*B. retamae* Ro19^T^ (LLYA00000000)	99.0	85.0	84.8	29.2
15	*B. tropiciagri* SEMIA 6148^T^ (LFLZ00000000)	99.0	80.9	75.4	24.5
16	*B. uaiense* UFLA03-164^T^ (VKHP00000000)	99.8	80.8	76.3	24.5
17	*B. viridifuturi* SEMIA 690^T^ (LGTB00000000)	99.3	80.9	77.0	24.3
18	*B. yuanmingense* CGMCC 1.3531^T^ (VLKS00000000)	98.1	79.3	74.9	22.9

Within the genus *Bradyrhizobium*, phylogenetic analysis based on MLSA (multilocus sequence analysis) of housekeeping genes is a powerful tool for discriminating species (Rivas et al., 2009; Tang et al., 2012; Duran et al., 2014). The phylogenetic tree of five concatenated housekeeping gene sequences (*ITS*, *dnaK*, *glnII*, *recA*, and *rpoB*) located the novel strain in a clearly distinct lineage from the named *Bradyrhizobium* species. The phylogenetic tree of the concatenated *ITS-dnaK*-*glnII*-*recA*-*rpoB* gene sequences confirmed the placement of the novel strain in a lineage distinct from the named *Bradyrhizobium* species (Supplementary Figure 3). ANI, AAI, and dDDH analyses based on whole-genome sequences were implemented for additional characterization. We estimated the values for the complete genome sequence of S12-14-2 by conducting pair-wise comparisons with genome sequences of the type strains of *Bradyrhizobium* species obtained from public databases. The ANI values of the named species ranged from 79.2% (*Bradyrhizobium daqingense*) to 86.6% (*B. algeriense*), which is significantly lower than the 95–96% threshold value for circumscription of bacterial species (Konstantinidis and Tiedje, 2005; Rivas et al., 2009; Chun et al., 2018; Ciufo et al., 2018). The AAI (dDDH) values for all pairwise comparisons between the novel strain and related *Bradyrhizobium* species ranged from 71.6 to 86.5% (22.8–32.5%) (Table 1), which is also under the borderline of 95% (ANI) and 70% (dDDH) species delineation (Goris et al., 2007; Richter and Rossello-Mora, 2009; Luo C. et al., 2014; Konstantinidis et al., 2017).

### General genome properties

The genome of isolate S12-14-2 has a size of 7,319,411 base pairs, a GC content of 63.3%, three rRNA genes, 62 tRNA genes, and 6893 CDS, of which 5,838 genes were allocated to COG (Supplementary Table 3). *Bradyrhizobium* spp. have relatively large genomes compared to other groups, ranging between 5 and 11M, whereas the smallest genome size found to date is approximately 5M in the free-living strain S2-11-2 (Ormeño-Orrillo and Martínez-Romero, 2019; Jin et al., 2022b; Klepa et al., 2022; Zhang et al., 2023). The GC content is consistent with other *Bradyrhizobium* type strains (Figure 1 and Supplementary Table 2). The abundant genes were associated with amino acid transport and metabolism (COG category E), inorganic ion transport and metabolism (COG category P), energy production and conversion (COG category C), transcription (COG category K), signal transduction mechanisms (COG category T), and cell wall/membrane/envelope biogenesis (COG category M), and lipid transport and metabolism (COG category I), and approximately 32.5% of the genes were assigned to the unknown function COG category (Supplementary Table 4).

### Pangenomics and secondary metabolites

To investigate the genomic diversity of *Bradyrhizobium* sp. S12-14-2 and related *Bradyrhizobium* spp., we compiled a total of 44 complete whole genomes (including 7 free-living and 34 symbiotics) downloaded from the NCBI. This dataset contains genomes representing 41 species validly published. Across the entire dataset, the genome size (GS) varied between 5.5 and 11.5M (mean, 8.5M); the GC content varied between 61.4 and 65.1% (mean, 64.9), and the number of predicted genes ranged from 5,220 to 11,061 (mean, 8,111).

**FIGURE 2:**
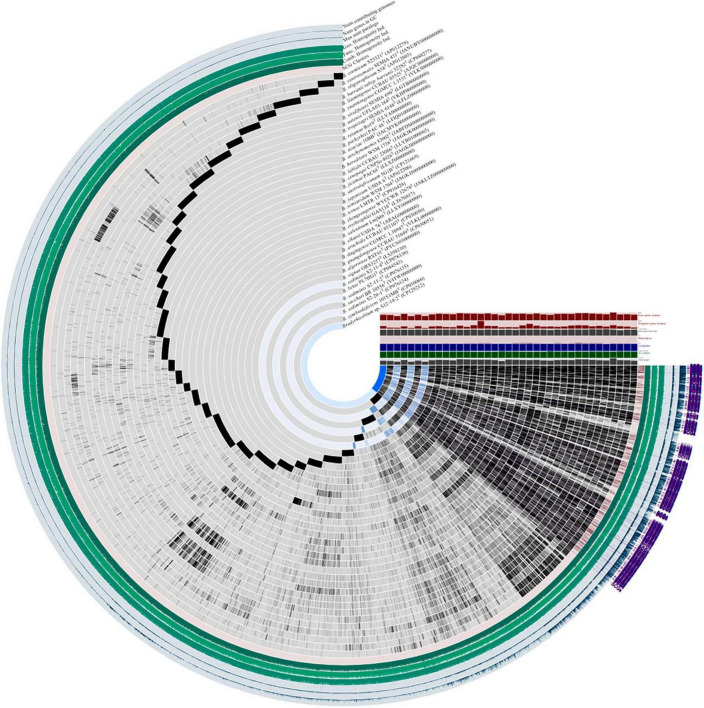
Pangenome analysis of *Bradyrhizobium* species including *Bradyrhizobium* sp. S12-14-2^T^ and closely related strains calculated with Anvi’o version 7.1. Dark regions in purple color (SCGs) represent genes (black) found in each genome at least one time.

Following the workflow, pangenomic analysis of 37 *Bradyrhizobium* strains was performed using Anvi’o (version 7.1). Single copy gene clusters (SCGs) are shown in purple color on the outside, which are drawn by 1,235 gene clusters from 43,916 splits and 44,526 objects. *Bradyrhizobium* sp. S12-14-2*^T^* exhibited a similar pattern of blue-colored single copy gene clusters as the other three free-living microorganisms *Bradyrhizobium sediminis* S2-20-1*^T^*, *B. sediminis* S2-11-2, and *B. sediminis* S2-11-4. SCGs were also the same with the core genes, and others not included in the SCGs were accessory genes. *B. erythrophlei* GAS138*^T^* (LT670817) has the largest number of singleton gene clusters which is almost equal to 2,046 (Figure 2). BGC from 44 *Bradyrhizobium* genomes from the free-living and symbiotic groups were identified using antiSMASH, and a comprehensive network analysis was performed in BiG-SCAPE using all similar BGC sequences from the MIBiG databases. The average number of BGCs from the 44 genomes is 11, and the network analysis distributed 557 BGCs in different types, including 99 terpenes, 84 NRPS (non-ribosomal peptides), 93 hserlactones (homoserine lactones), 44 redox-cofactors (redox-cofactors such as PQQ), 47 RiPP-like (other unspecified ribosomally synthesized and post-translationally modified peptide products), 35 NRPS-like (NRPS-like fragmenta), and 37 TPKS (type I, II, and III PKSs), and generally, the number of secondary metabolites from free living bacteria are less than symbiotic bacteria (Supplementary Figure 4). We identified common BGCFs shared by all strains, such as terpenes and redox-cofactors and nine BGCFs not found in free-living strains, such as *Bradyrhizobium* sp. S12-14-2 (Supplementary Figure 4). Additionally, *Bradyrhizobium* sp. S12-14-2 possesses a phosphonate-producing gene cluster, confirmed by phosphorous metabolism-related genetic analysis.

### Genome size of *Bradyrhizobium*

Genome size variation is a biological trait, although its evolutionary sources and effects are debated. GS’s neutrality, selective pressures, and strength are unknown. The genetic segments responsible for this diversity directly affect evolutionary outcomes and are targeted by diverse evolutionary pressures. *Bradyrhizobium* isolates have rather big genomes ranging from 5 to 11M, with an average size of 8.6M (Ormeño-Orrillo and Martínez-Romero, 2019; Avontuur et al., 2022; Jin et al., 2022b; Claassens et al., 2023; Lafay et al., 2023; Zhang et al., 2023). Nevertheless, the range of GSs within the genus remains unknown. The smallest genome was 5.5M in length and related to strain S12-14-2 recovered from freshwater sediment. The largest full assembly (11.7M) was discovered from the soil isolate GAS478 (FSRD01000000). Notably, strains S12-14-2 (7.3M), S2-20-1*^T^* (5.6M), S2-11-2 (5.5M), S2-11-4 (5.5M), and GAS478 (11.7M) lacked symbiosis genes, suggesting that GS in *Bradyrhizobium* is unrelated to its capacity to interact with legumes. In the *Nitrobacteraceae* family, *Bradyrhizobium* has the largest genome, followed by *Tardiphaga* and *Bosea*. *Bradyrhizobium*, *Tardiphaga*, and *Bosea*, three taxa with large genomes, interact with plants (De Meyer et al., 2012; Ormeño-Orrillo and Martínez-Romero, 2019). And taxa with small genomes, including *Nitrobacter*, *Oligotropha*, and *Variibacter*, were discovered to be metabolically constrained, indicating that the *Nitrobacteraceae* GS appears to be associated with lifestyle. Understanding the GS variation and its causes and effects requires trustworthy methodologies; however, there are still obstacles. From genome annotation and content analysis to laboratory- and sequence-based GS measurement accuracy, there are problems and recommended practices. Even among big consortia building high-quality genome assemblies, gold standards and best practices are continually changing.

### Motility

The genome of strain S12-14-2 contains 192 genes for motility via flagella and genes for chemotaxis (data not shown). At least 132 genes related to flagellar motility (components of the flagellar apparatus or specific regulators) are grouped in two clusters in the genome separated by unknown genes. Notably, the cultured S12-14-2 cells lack any discernible flagella or pili (Supplementary Figure 1). The reason could be that twitching motility may be a more efficient mode of locomotion in environments devoid of conditions that require or prohibit pilus-facilitated or flagellum-powered movement.

### Genes involved in carbon metabolism and photosynthesis

**FIGURE 3:**
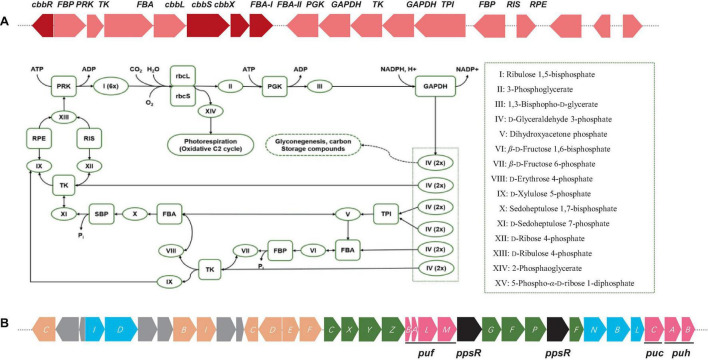
**(A)** Diagram of Calvin cycle and RuBisCO gene cluster arrangements including RuBisCo (RbcL), phosphoribulokinase (PRK), fructose-1,6-bisphosphatase (FBP), etc. Color keys: red, RuBisCO subunits and RuBisCo activation protein coding genes: rose, other carbon fixation related genes. **(B)** Photosynthetic gene cluster of *Bradyrhizobium* sp. S12-14-2^T^. Color keys: green, *bch* genes (bacteriochlorophyll synthesis); orange, *crt* genes (carotenoid synthesis); blue, *chl* gene (light-independent protochlorophyllide reductase); pink, *puh* (photosynthetic reaction centers), *puf* and *puc* (light-harvesting complexes); black, regulatory proteins; gray, uncharacterized proteins.

Free-living *Bradyrhizobium* organisms also perform vital roles in soil ecology, nutrient cycling, and carbon metabolism. As free-living microbes, they must acquire carbon from other environmental sources. *Bradyrhizobium* can metabolize a vast array of carbon sources, including simple carbohydrates, organic acids, and amino acids, via diverse biochemical pathways (VanInsberghe et al., 2015; Tao et al., 2021). The TCA (tricarboxylic acid) cycle, also known as the Krebs cycle, is one of the primary pathways involved in carbon metabolism in free-living *Bradyrhizobium*. This pathway is essential for the growth and survival of microorganisms as it is involved in producing energy from carbon sources. The complete gene sets of the TCA cycle, Entner–Doudoroff pathway, and glycolysis and gluconeogenesis were observed in the genome of strain S12-14-2. Furthermore, genes for encoding phosphoenolpyruvate (PEP) carboxylase (EC 4.1.1.31) and pentose phosphate pathway were also observed. The genome of S12-14-2 contained genes for encoding RuBisCO (ribulose 1,5-bisphosphate carboxylase/oxygenase) or the light-harvesting complex (Figure 3A), which means that strain S12-14-2 may fix CO_2_ and potentially have photosynthetic function.

The gene cluster responsible for photosynthesis in *Bradyrhizobium* encompasses several genes that have a crucial role in the production of pigments and proteins necessary for the process of photosynthesis. The genes in question encode various proteins, such as bacteriochlorophyll, carotenoids, and reaction center proteins (Gregor and Klug, 1999; Igarashi et al., 2001; Hata et al., 2023). The presence of genes associated with photosynthesis in the genome of S12-14-2 has been verified. The genetic components in this set are as follows: bacteriochlorophyll genes (*bchCXYZGFPF*), genes for light-independent protochlorophyllide reductase (*chlIDNBL*), carotenoid genes (*crtCBICDEF*), genes for light harvesting polypeptides (*pucCAB*), and genes for reaction center subunits (*puhBA/pufLM*) (Figure 3B). Several species of *Bradyrhizobium* have the ability to utilize carbon dioxide through the Calvin cycle, also known as the Calvin–Benson–Bassham cycle. In this metabolic pathway, the enzyme ribulose-1, 5-bisphosphate carboxylase oxygenase (RuBisCo) has a crucial role. The strain S12-14-2 was found to possess all gene sets associated with the Calvin cycle except for a gene responsible for encoding sedoheptulose-1,7-bisphosphatase (SBP) (Figure 3A). The Calvin cycle involves the execution of distinct tasks by RuBisCo (RbcL), phosphoribulokinase (PRK), and SBP. The combined utilization of RuBisCo and PRK can be used as reliable indicators for the presence of the cycle within a particular organism. However, the enzymatic activity of SBP in bacteria is commonly facilitated by fructose-1,6-bisphosphatase (FBP) with a dual sugar (Jiang et al., 2012).

### Genes involved in nitrogen metabolism and nodulation

**FIGURE 4:**
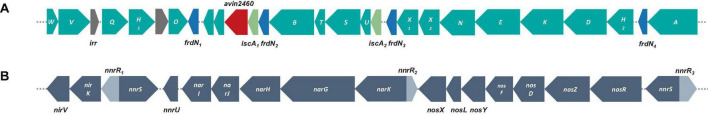
Genetic organization of nitrogen-fixation and denitrification related genes and surrounding genes. **(A)** Nitrogen-fixation gene clusters located on genome of strain S12-14-2^T^. **(B)** Denitrification gene clusters; color keys: teal, *nif* genes; gray, *irr* genes (iron-responsive regulator); blue, *frdN* genes (4Fe-4S ferredoxin, nitrogenase-associated); red, *avin2460* [LRV (FeS)4 cluster domain protein clustered with nitrogenase cofactor synthesis]; light green, *iscA* genes (probable iron binding protein in *nif* operon); gray, denitrification related genes: *nor*, nitric oxide reductase; *nar*, nitrate reductase; *nir*, nitrite reductase; *nnr* genes [*nnrS* (NO sensing protein); *nnrU* (denitrification regulatory protein)].

Nitrogen fixation is extremely important in environmental sustainability, particularly in nitrogen-deficient soil (Lindström et al., 2010; Hungria et al., 2015). In legume-rhizobium symbiosis, this activity is key for soil health and plant growth (Jensen and Hauggaard-Nielsen, 2003; Meena et al., 2018). Genes encoding NifKDH (nitrogenase α-, ß-subunits and reductase, respectively); and NifENB (nitrogenase assembly proteins) are considered the minimal gene set required for a diazotrophic phenotype; those genes encode a FeMo-cofactor-containing nitrogenase (Kaneko et al., 2002; Dos Santos et al., 2012; Sprent et al., 2017). All genes encoding these nitrogen-fixation like proteins in S12-14-2 (genes and proteins abbreviated *nif* and Nif, respectively, and numbered 1–2), *nifABDEHKNOQSTUVWXZ*, were clustered (Figure 4A). The genes encoding NifH_1_,_2_DK are located upstream of the cluster, while the genes encoding NifNE are adjacent to *nifKDH*. In contrast to symbiotic strains, free-living *Bradyrhizobium* bacteria do not have *nif* or *nod* genes, and some nod-independent strains contain just *nif* genes (Ferrieres et al., 2004; Bobik et al., 2006; Meilhoc et al., 2010; VanInsberghe et al., 2015). No nodulation genes were identified in the genome, indicating that strain S12-14-2 could fix atmospheric nitrogen; as for nodulation, it is uncertain whether strain S12-14-2 forms nodules at this time because of nod-factor-independence of symbiosis.

Denitrification is a prominent contributor to nitrous oxide (N_2_O) emission in soil, serving as a substantial constituent of the worldwide nitrogen cycle. This process takes place in both terrestrial and marine ecosystems. Certain bacteria can adjust their metabolic processes in response to oxygen-depleted settings, wherein the concentration of dissolved and accessible oxygen is diminished. In such conditions, these bacteria use nitrate as a respiratory substrate for the process of denitrification (Coates et al., 2001; Luo F. et al., 2014; Zhang et al., 2020). The strain S12-14-2 contains a periplasmic nitrate reductase (Nar), a copper (Cu)-containing nitrite reductase (Nir), and a Cu-dependent nitrous oxide reductase (Nos) encoded by the *narGHJI, nirKV*, and *nosRZDFYLX* genes, respectively (Figure 4B). Nitrite produced by NarGHJI will be released to the periplasm through a membrane transporter and reduced to nitric oxide (NO) by Cu-containing nitrite reductase (NirK) (Richardson, 2000; Simon and Klotz, 2013). Due of its high cytotoxicity, NO will immediately reduce to N_2_O by Nor (nitric oxide reductase). Typically, nor enzymes are usually periplasmic cytochrome *c* (cNor) or quinones (qNor) (Nordling et al., 1990; Pearson et al., 2003; Rinaldo and Cutruzzola, 2007; Simon and Klotz, 2013). Notably, the genome does not contain NO reductase genes for the reduction stage. The apparent absence of Nor-encoding genes in strain S12-14-2 is of special interest, but it is speculated that some cNor or qNor-like proteins are encoded by some other unknown genes.

### Genes involved in sulfur metabolism

**FIGURE 5:**
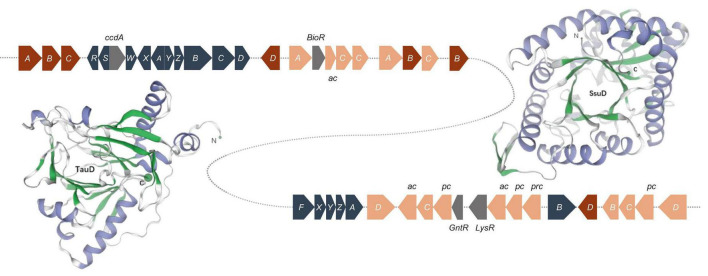
Sulfur oxidation gene clusters and of structure of the monomeric unit of TauD and SsuD. The TauD and SsuD enzyme exist in the form of a TIM-barrel containing 297 and 359 amino acid residues, respectively. Color key: blue gray, *sox* genes; red, *tau* genes; orange, *ssu* genes (*ac*, ABC type nitrate/sulfonate/bicarbonate transport system, ATPase component; *pc*, ABC-type nitrate/sulfonate/bicarbonate transport systems, periplasmic components; *prc*, ABC-type nitrate/sulfonate/bicarbonate transport system, permease component.); gray, regulator genes.

Sulfur, an amino acid component and enzyme cofactor, is essential to all organisms. Sulfide is formed by the sulfate assimilation pathway in many bacteria and integrated into sulfur-containing organic compounds. Under sulfur-limiting conditions, bacteria must receive sulfur from the environment because inorganic sulfate is not prevalent in nature. Sulfonates and sulfonate esters are natural or xenobiotic compounds (Kertesz, 2000; Van Der Ploeg et al., 2001b). Genes encoding a sulfur-oxidizing (Sox) function were discovered, and the *sox* gene cluster consists of 16 genes. *soxR* encodes a repressor protein, and *soxSW* encodes a periplasmic thioredoxin, which is essential for full expression of the *sox* genes. The subsequent *soxXYZABCD* genes encode four periplasmic proteins that reconstitute the Sox enzyme system: SoxXA, SoxYZ, SoxB, and SoxCD, which can convert a variety of reduced sulfur compounds to sulfates (Figure 5). Strain S12-14-2 also possess the *soxF* gene, which encodes flavoprotein SoxF with a sulfide dehydrogenase activity (Quentmeier et al., 2004). These sulfur oxidation Sox systems suggest that strain S12-14-2 might be capable of oxidizing thiosulfate to sulfate.

Under sulfur-limiting conditions, several bacteria express proteins to use organosulfonates or sulfonate esters as sulfur sources. Sulfate starvation-induced proteins ingest sulfonate, acquire sulfur from organic molecules, and defend against reactive oxygen species (Hummerjohann et al., 1998; Van Der Ploeg et al., 2001a; Ellis, 2011). A sulfonate transport system has been detected in the genome of *Bradyrhizobium* sp. S12-14-2, which includes sulfonate-binding protein (SsuA), ATP-binding protein (SsuB), and sulfonate permease protein (SsuC). The genes *tauD* and *ssuD*, which encode a taurine dioxygenase and an alkanesulfonate monooxygenase, respectively, were also found to be responsible for sulfonate and taurine metabolism. The *tau* operon encodes an ABC-type transporter and taurine dioxygenase (TauD), which oxidize taurine to hydroxytaurine. The unstable hydroxytaurine breaks down to yield the aminoacetaldehyde and sulfite products (Eichhorn et al., 1997). The SsuD monooxygenase enzyme catalyzes the desulfonation of a wide variety of organosulfonated products, producing the corresponding aldehyde and sulfonate (Eichhorn et al., 1999).

The structures of TauD and SsuD monomeric units were predicted using the SWISS-MODEL. The SsuD monomer consists of a total of 359 amino acid residues and belongs to the structural family of TIM-barrel proteins. SsuD is classified as a flavin-dependent oxidoreductase belonging to the LLM (luciferase-like monooxygenase) class. On the other hand, the TauD monomer consists of 297 amino acid residues and functions as an α-ketoglutarate-dependent dioxygenase (Figure 5). TauD prefers taurine but may also desulfonate alkanesulfonates. Although alkanesulfonate monooxygenase enzymes can use various organosulfate substrates, they have no enzymatic activity against taurine (Ellis, 2011). These TauD and SsuD enzymes possibly work together to ensure that the bacterial cell has sufficient sulfur for biosynthetic processes. While most of these enzymes’ kinetic properties are known, much remains to be learned about their detailed mechanistic properties.

### Genes involved in phosphate metabolism

**FIGURE 6:**
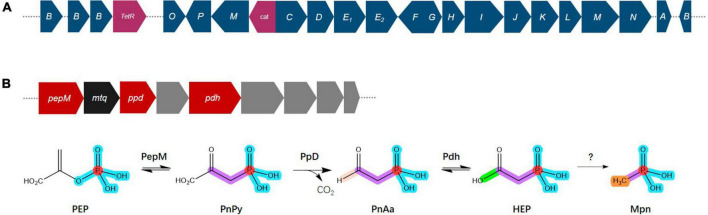
Overview of the C–P lyase gene cluster and pathway. **(A)** Overview of *phn* gene organization. **(B)** Pathway for phosphonate production and gene organization. Phosphoenolpyruvate (PEP) mutase (PepM) isomerizes PEP to phosphonopyruvate (PnPy), which is the precursor to all known phosphonate molecules. Due to this functional conservation, the *pepM* gene is a reliable genomic marker for the potential to produce phosphonates. Phosphonopyruvate decarboxylase (Ppd) converts PnPy to phosphonoacetaldehyde (PnAa), phosphonoacetaldehyde dehydrogenase (Pdh) dehydrogenates of PnAa to 2-hydroxyethylphosphonic acid (HEP), and finally to methylphosphonate (Mpn). Color key: dark blue, *phn* genes; purple, *TelR* (transcriptional regulator), *cat* (chloramphenicol acetyltransferase); red, phosphonate production related genes; black, *mtq* (O-methyltransferase); gray, transporters.

Phosphorus is an essential nutrient for all organisms and has crucial roles in respiration, energy production and storage, the biosynthesis of nucleic acids, ATP, and phospholipids and other physiological and biochemical processes in specific environments (Balemi and Negisho, 2012; Nath et al., 2017). Phosphorus availability and plant assimilation are governed by microorganisms, and phosphorus-solubilizing microorganisms are capable of dissolving insoluble phosphorus in soil and converting it into soluble phosphorus for plant absorption and use (Cao, 2014; Moro et al., 2021). Phosphonate compounds, which contain a direct C–P bond instead C–O–P ester linkage, are prevalent in the environment, where they are a significant source of organic phosphorus and a common pollutant (Horsman and Zechel, 2017; Rott et al., 2018). The degradation of phosphonates is especially advantageous for microorganisms, where the bioavailability of phosphorus is frequently a growth-limiting factor (Sosa et al., 2019). The genome of *Bradyrhizobium* sp. S12-14-2 contains all the predicted C–P lyase genes necessary for phosphonate degradation and the additional putative auxiliary functions (Figure 6A). These genes included a *TetR* family transcriptional regulator, phosphonate ABC transporters *phnCDE_1_E_2_*, purine ribonucleoside triphosphate phosphorylase components *phnGHIJKL*, a chloramphenicol acetyltransferase, a triphosphoribosyl 1-phosphonate diphosphohydrolase *phnM*, and a phosphoribosyl cyclic phosphodiesterase *phnP*.

Phosphonates are a type of phosphorus-based metabolites distinguished by a highly stable C–P bond. They comprise a significant portion of the dissolved organic phosphorus reservoir in the oceans and are widely distributed among more primitive life forms (Villarreal-Chiu et al., 2012; Acker et al., 2022). Almost all known pathways of phosphonate biosynthesis involve a central C–P bond-forming reaction in which the enzyme PEP phosphomutase (Ppm) intramolecularly rearranges the intermediary metabolite phosphoenolpyruvate (PEP) to form phosphonopyruvate (PnPy) (Metcalf and Van Der Donk, 2009; Acker et al., 2022). The genome of *Bradyrhizobium* sp. S12-14-2 contains the genes *pepM* which encodes Ppm, *ppd* encoding phosphonopyruvate decarboxylase (Ppd) which subsequently converts PnPy to phosphonoacetaldehyde (PnAa) in next reaction, and *pdh* encoding phosphonoacetaldehyde dehydrogenase (Pdh) which dehydrogenates of PnAa to 2-hydroxyethylphosphonic acid (HEP) (Figure 6B).

## Conclusion

To summarize our investigations, the strain S12-14-2^T^ from a contaminated freshwater sediment belonging to the genus *Bradyrhizobium* was studied through genome analysis and a polyphasic approach. The phylogenetic analysis indicated that the strain S12-14-2^T^ was affiliated closely with the species of *Bradyrhizobium*, based on the phylogenetic, genomic, and physiological differences; thus, we propose strain S12-14-2^T^ as a novel species, *Bradyrhizobium roseus* sp. nov., in the family *Nitrobacteraceae* (Table 2). The *B. roseus* pan-genome is considered to be in an open state, genetically diverse, and with a flexible gene repertoire associated with multiple functions. *B. roseus* contained genes encoding all enzymes of the Calvin–Benson cycle and phototrophic systems with carotenoids synthesizing genes and possesses complete genes for nitrogen fixing and denitrification. *B. roseus* S12-14-2^T^ also contains genes for sulfur oxidation and the essential genes encoding for the sulfonate transport system. Moreover, strain S12-14-2^T^ contains all the necessary genes for phosphonate degradation and phosphate biosynthesis. The physiological characteristics and comparative genome analyses of *B. roseus* S12-14-2^T^ help us to understand the metabolism and evolutionary features of the genus *Bradyrhizobium* and provide understanding of this microorganism and a new genetic framework for future studies.

**TABLE 2:** Descriptions of *Bradyrhizobium roseus* sp. nov.

Genus name	*Bradyrhizobium*
Species name	*Bradyrhizobium roseus*
Species epithet	*roseus* sp. nov.
Species etymology	ro′se.us. L. masc. adj. *roseus*, rose colored, pink
Description of the new taxon and diagnostic traits	Cells are Gram-stain-negative and rods. Colonies grown on R2A agar are pink. Growth occurs on R2A at temperatures from 15 to 30°C (optimum temperature 25–30°C) but not 10 or 37°C. The pH range for growth is from pH 6.0 to 9.0 and optimum pH 7–8 but not at pH 5.0 and 10.0. Cells are found to be positive for nitrate reduction and indole production, but negative for glucose acidification, urease, esculin hydrolysis, gelatin hydrolysis, and β-galactosidase. Cells utilize L-proline and L-serine but not acetate, N-acetyl glucosamine, adipate, L-alanine, L-arabinose, caprate, citrate, L-fucose, gluconate, D-glucose, glycogen, histidine, 3-hydroxybenzoate, 4-hydroxybenzoate, 3-hydroxybutyrate, inositol, itaconate, 2-ketogluconate, 5-ketogluconate, lactate, malate, malonate, D-maltose, D-mannitol, D-mannose, D-melibiose, phenylacetate, propionate, L-rhamnose, D-ribose, salicin, D-sorbitol, suberate, D-sucrose, or valerate. Cells are found to be positive for the following enzyme activities: alkaline phosphatase, esterase (C4), esterase lipase (C8), leucine arylamidase and naphthol-AS-BI-phosphohydrolase (weakly). Cells are found to be negative for the following enzyme activities: *N*-acetyl-β-glucosaminidase, acid phosphatase, α-chymotrypsin, cystine arylamidase, α-fucosidase, α-galactosidase, β-galactosidase, α-glucosidase, β-glucosidase, β-glucuronidase, lipase (C14), α-mannosidase, trypsin, and valine arylamidase. The major fatty acids are grouped into two categories C_18:1_ ω7*c* and/or C_18:1_ ω6*c* and C_16:0_.
Region of origin and sampling date (mm/yyyy)	Hongze Lake, China (10/2018)
Latitude (xx°xx′xx″N/S, xx°xx′xx″E/W)	33°14′26″N, 118°35′40″E
The GenBank accession number for 16S rRNA gene and genome	MZ315001, CP129212 (complete genome)
Genome size (GC content)	7,319,411 bp (63.3%)
Designation of the type strain and isolation source	S12-14-2^T^ isolated from freshwater sediment
Strain collection numbers	JCM 34606^T^; CGMCC 1.19422^T^

## Data availability statement

The datasets presented in this study can be found in online repositories. The names of the repository/repositories and accession number(s) can be found in the article/Supplementary material.

## Author contributions

NZ: Conceptualization, Data curation, Writing – original draft. C-ZJ: Conceptualization, Data curation, Writing – original draft. YZ: Data curation, Validation, Visualization, Writing – original draft. TL: Data curation, Validation, Visualization, Writing – original draft. F-JJ: Data curation, Validation, Visualization, Writing – original draft. H-GL: Resources, Writing – original draft, Writing – review and editing. LJ: Resources, Supervision, Writing – original draft, Writing – review and editing.

## Footnotes

1
http://www.ncbi.nlm.nih.gov/COG/

2
https://antismash.secondarymetabolites.org

3
http://enve-omics.ce.gatech.edu/aai/
