# Supplementary material for: Genetic diversity into a novel free-living species of Bradyrhizobium from contaminated freshwater sediment
Source: Front Microbiol. 2023 Nov 18;14:1295854. doi: 10.3389/fmicb.2023.1295854 (PMC10708946; doi:10.3389/fmicb.2023.1295854)

Supporting Material

Genetic diversity into a novel free-living species of *Bradyrhizobium* from contaminated freshwater sediment

Naxue Zhang[1, ‡], Chun-Zhi Jin[2, ‡], Ye Zhuo[2, 3], Taihua Li[1], Feng-Jie Jin[1], Hyung-Gwan Lee[2, *], Long Jin[1, *]

[1] College of Ecology and Environment, Nanjing Forestry University, Nanjing 210-037, China

[2] Cell Factory Research Centre, Korea Research Institute of Bioscience & Biotechnology (KRIBB), Daejeon 34141, Republic of Korea

[3] Department of Environmental Biotechnology, KRIBB School of Biotechnology, Korea University of Science and Technology (UST), Daejeon, Republic of Korea

Running title: Genetic diversity into a novel free-living species of *Bradyrhizobium*

‡ Both authors contributed equally to this work

*** Corresponding authors**

Long Jin

Tel: +86-25-8542-7210

E-mail: isacckim@alumni.kaist.ac.kr

Hyung-Gwan Lee

Tel: +82-42-860-4318

E-mail: trustin@kribb.re.kr

Supplementary Table S1. Cellular fatty acid compositions (%) of strain S12-14-2^{T} and the type strains of related species of the genus *Bradyrhizobium*.

Strains: 1, S12-14-2^{T}; 2, *B. sediminis* S2-20-1^{T}; 3, *B. erythrophlei* LMG 28425^{T}; 4, *B. jicamae* LMG 24556^{T}. 5, *B. lablabi* LMG 25572^{T}; 6, *B. mercantei* LMG 30031^{T}; 7, *B. elkanii* KACC 10647^{T}; 8, *B. japonicum* KACC 10645^{T}. Cells of all strains were harvested after growth on R2A agar at 28 °C for 3 days. –, not detected.

Fatty Acids	**1**	**2**	**3**	**4**	**5**	**6**	**7**	**8**
$C_{16:1}$ *ω*9*c*	1.6	–	–	–	–	–	–	–
$C_{16:0}$	16.8	19.3	15.8	13.8	18.2	23.1	16.5	17.7
$C_{17:1}$ *ω*8*c*	–	–	–	–	1.4	–	–	–
$C_{17:1}$ *ω*6*c*	–	–	–	–	1.5	–	–	–
$C_{17:0}$ cyclo	–	–	2.0	–	–	3.5	1.8	–
$C_{17:0}$	–	–	–	–	0.9	–	–	–
$C_{18:0}$	–	–	–	1.1	0.6	–	0.5	–
$C_{18:1}$ *ω*7*c* 11-methyl	5.3	–	–	2.6	1.8	–	–	12.6
$C_{19:0}$ cyclo *ω*8*c*	–	–	9.3	–	–	31.9	40.1	–
$C_{20:2}$ *ω*6, 9*c*	–	–	–	–	–	–	0.8	–
$C_{20:1}$ *ω*7*c*	–	–	–	–	–	–	–	–
Summed Feature 3‡	2.1	14.0	2.9	1.6	2.2	–	0.5	2.1
Summed Feature 8§	74.2	66.7	70.0	80.9	73.5	41.5	39.8	67.7

Note: ‡Summed Feature 3 contains $C_{16:1}$ *ω*7*c* and/or $C_{16:1}$*ω*6*c*; §summed feature 8 contains $C_{18:1}$ *ω*7*c* and/or $C_{18:1}$ *ω*6*c*.

Supplementary Table S2. Comparative characteristics of strain S12-14-2^T from some close species of *Bradyrhizobium*.

Strains: 1, *B. roseus* S12-14-2^T; 2, *B. sediminis* S2-20-1^T; 3, *B. erythrophlei* LMG 28425^T; 4, *B. jicamae* LMG 24556^T. 5, *B. lablabi* LMG 25572^T; 6, *B. mercantei* LMG 30031^T; 7, *B. elkanii* KACC 10647^T; 8, *B. japonicum* KACC 10645^T. Data are from the present study unless indicated. +, positive; –, negative; w, weakly positive.

**Characteristics**	**1**	**2**	**3**	**4**	**5**	**6**	**7**	**8**
Urease	+	–	–	–	+	+	+	–
Carbon utilization :								
L-Arabinose	–	–	–	+	+	+	+	+
Citrate	–	–	+	–	+	–	+	+
Gluconate	–	–	+	–	–	–	–	–
D-Glucose	–	–	–	–	–	+	+	–
Maltose	–	–	–	–	–	w	–	–
D-Mannitol	–	+	+	–	+	+	+	+
D-Mannose	–	–	–	–	+	+	+	+
Enzyme activity :								
Alkaline phosphatase	+	+	–	–	+	–	+	–
α-Chymotrypsin	–	+	–	–	+	+	+	+
Cystine arylamidase	–	+	+	–	–	–	–	–
Esterase (C4)	+	+	–	+	+	+	+	+
Esterase Lipase (C8)	+	+	–	–	–	–	–	–
Leucine arylamidase	+	+	+	–	–	–	–	–
Lipase (C14)	–	–	+	–	–	–	–	–
Naphthol-AS-BI-phosphohydrolase	w	w	–	–	–	–	–	–
Trypsin	–	+	–	–	–	–	–	+
DNA G+C content (%)	63.3	63.6	62.0	64.1	60.1	64.0	63.7	63.7

Supplementary Table S3. The genome features of strain S12-14-2^{T}.

Attribute	Value	% of Total
Genome size (bp)	7,319,411	100
DNA coding (bp)	6,385,338	87.2
Total genes	6,963	100
Genes assigned to COG	5,838	83.8
Pseudo genes	89	1.3
RNAs	70	1.0
tRNAs	62	0.9
ncRNAs	5	0.1
rRNA genes (5S/16S/23S)	3 (1/1/1)	0.04
Gene island	7	0.1
CRISPR	2	0.03
Chromosome G + C content (%)	63.3	–

Supplementary Table S4. Distribution of genes in general COG categories of genome of strain S12-14-2^T.

Functional code	Description	Genes	Percentage
[U]	Intracellular trafficking, secretion, and vesicular transport	108	1.9
[D]	Cell cycle control, cell division, chromosome partitioning	27	0.5
[M]	Cell wall/membrane/envelope biogenesis	247	4.2
[V]	Defense mechanisms	68	1.2
[O]	Posttranslational modification, protein turnover, chaperones	203	3.5
[T]	Signal transduction mechanisms	263	4.5
[N]	Cell motility	74	1.3
[L]	Replication, recombination and repair	190	3.3
[B]	Chromatin structure and dynamics	2	0.0
[J]	Translation, ribosomal structure and biogenesis	189	3.2
[K]	Transcription	353	6.1
[F]	Nucleotide transport and metabolism	72	1.2
[P]	Inorganic ion transport and metabolism	410	7.0
[E]	Amino acid transport and metabolism	530	9.1
[Q]	Secondary metabolites biosynthesis, transport and catabolism	190	3.3
[H]	Coenzyme transport and metabolism	148	2.5
[I]	Lipid transport and metabolism	241	4.1
[C]	Energy production and conversion	372	6.4
[G]	Carbohydrate transport and metabolism	256	4.4
[S]	Function unknown	1896	32.5

Supplementary Figure S1. Transmission electron micrograph of strain S12-14-2^T grown on R2A medium for 3 days at 28 °C. Bar, 1 μm.

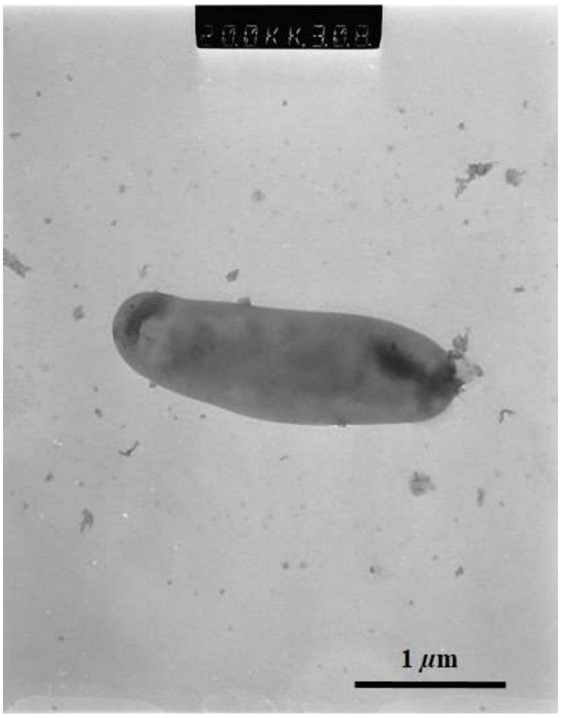

Supplementary Figure S2. Phylogenetic tree depicting the phylogenetic relationship between strain S12-14-2^{T} and other taxa based on 16S rRNA gene sequences using the neighbor-joining method in MEGA7. Numbers at bootstrap percentage based on 1000 replications (values over 50 % are shown). Bar, 1 substitution per 100 nt positions.

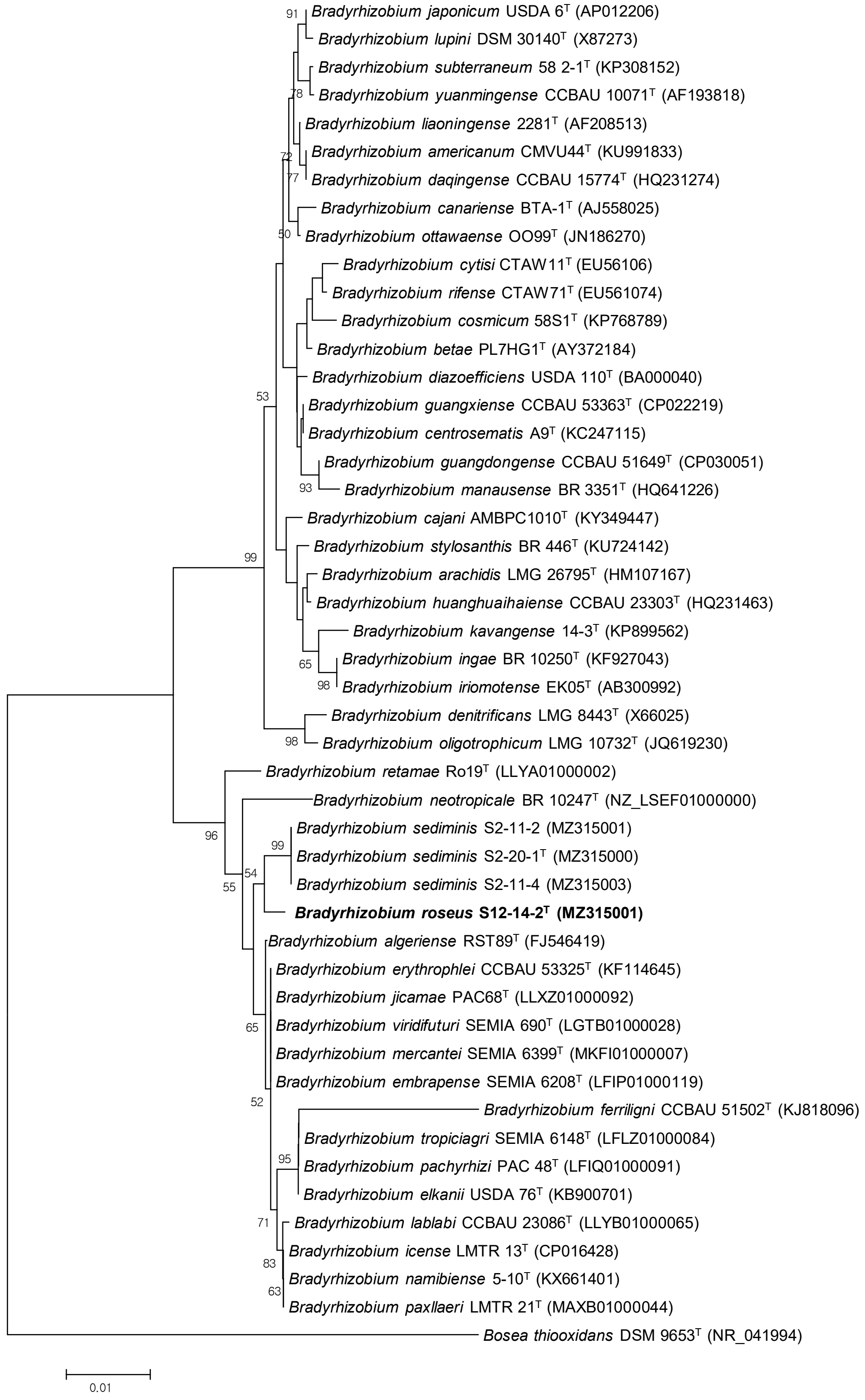

Supplementary Figure S3. Neighbor-joining phylogenetic tree based on concatenated *ITS+dnaK+glnII+recA+rpoB* gene sequences showing the position of strain S12-14-2^{T} among the type species within the genus *Bradyrhizobium*. Numbers at bootstrap percentage based on 1000 replications (values over 50 % are shown). Bar, 2 substitutions per 100 nt positions.

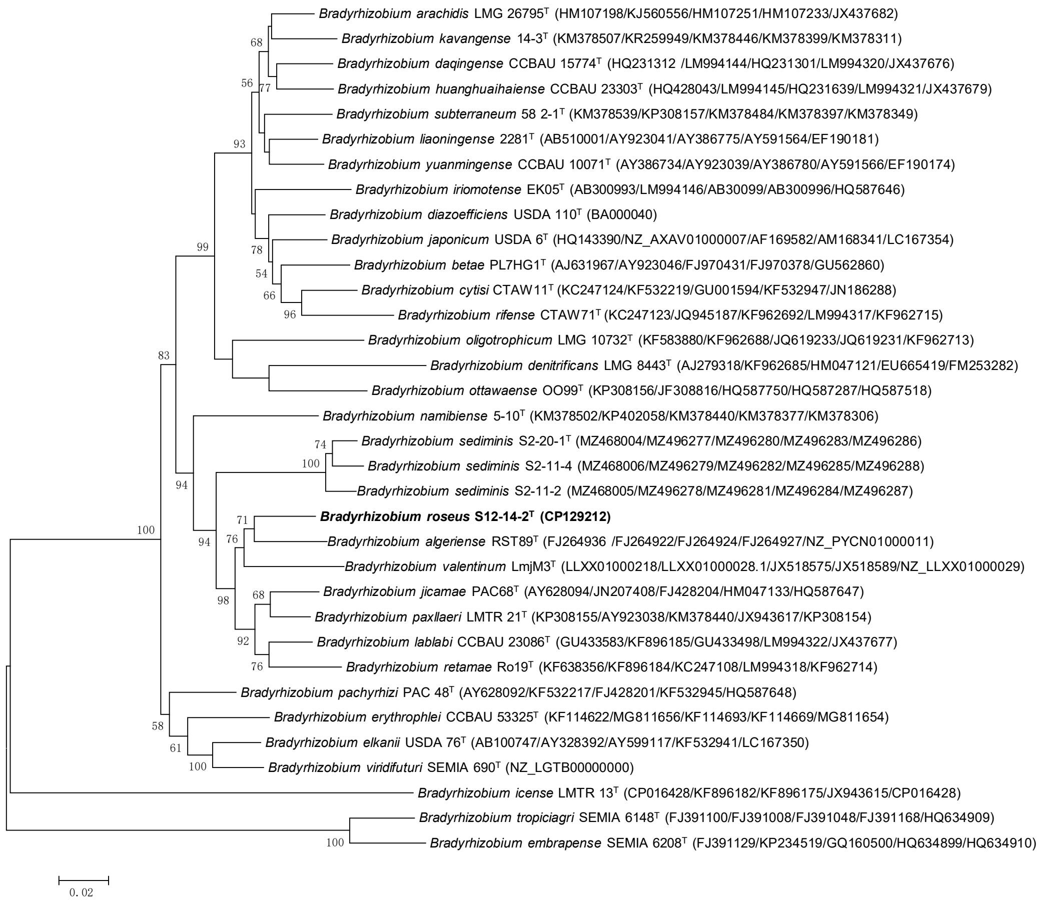

Supplementary Figure S4. Heat map of BGCs types from 44 genomes within the free-living and symbiotic groups identified in antiSMASH and BiG-SCAPE.

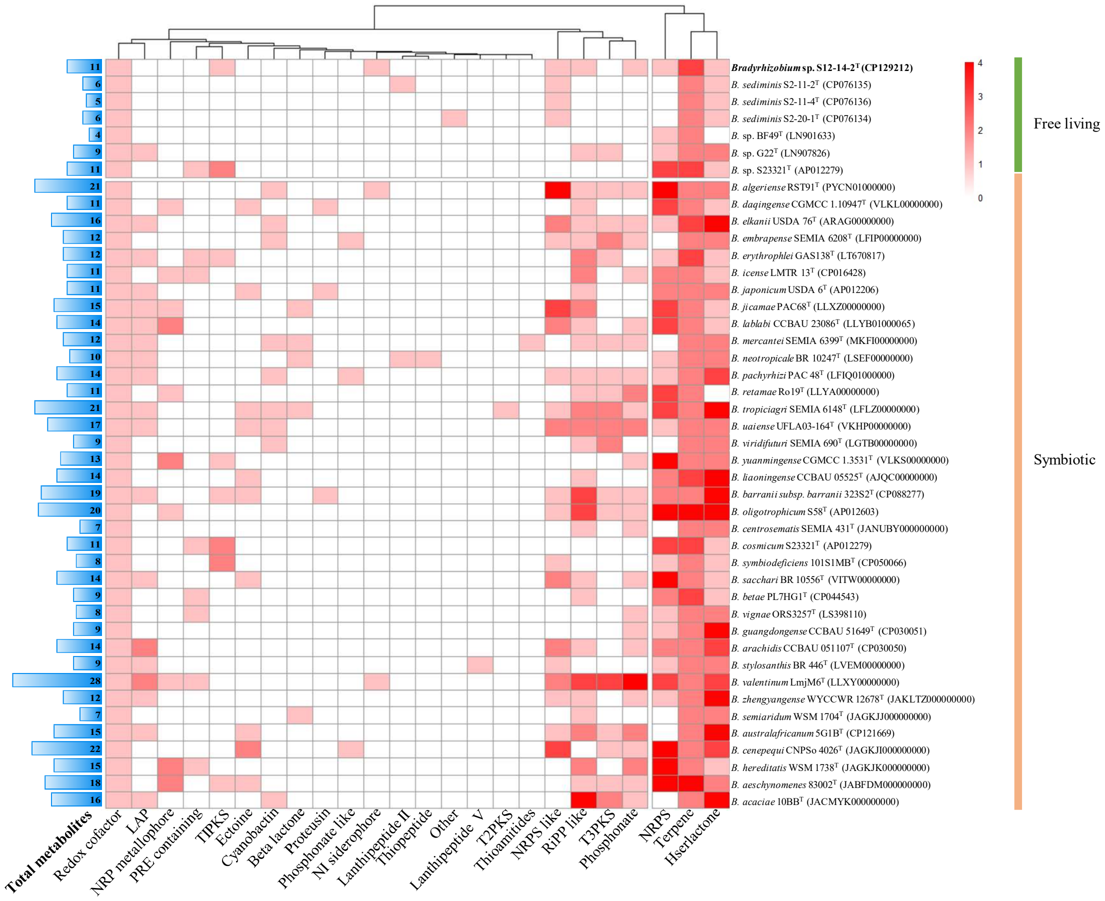

Supplement: Supplementary file 1 [file Data_Sheet_1.PDF]
